# Experimental Investigations on Airborne Gravimetry Based on Compressed Sensing

**DOI:** 10.3390/s140305426

**Published:** 2014-03-18

**Authors:** Yapeng Yang, Meiping Wu, Jinling Wang, Kaidong Zhang, Juliang Cao, Shaokun Cai

**Affiliations:** 1 Department of Automatic Control, College of Mechatronics and Automation, National University of Defense Technology, Deya Street 109, Changsha 410073, China; E-Mails: kdzhang@263.net (K.Z.); cjl.nudt@gmail.com (J.C.); csk527@163.com (S.C.); 2 School of Civil and Environmental Engineering, University of New South Wales, Sydney, NSW 2052, Australia; E-Mail: Jinling.Wang@unsw.edu.au

**Keywords:** compressed sensing, strap-down airborne gravimeter, airborne gravimetry, orthogonal matching pursuit, gravity anomaly data reconstruction

## Abstract

Gravity surveys are an important research topic in geophysics and geodynamics. This paper investigates a method for high accuracy large scale gravity anomaly data reconstruction. Based on the airborne gravimetry technology, a flight test was carried out in China with the strap-down airborne gravimeter (SGA-WZ) developed by the Laboratory of Inertial Technology of the National University of Defense Technology. Taking into account the sparsity of airborne gravimetry by the discrete Fourier transform (DFT), this paper proposes a method for gravity anomaly data reconstruction using the theory of compressed sensing (CS). The gravity anomaly data reconstruction is an ill-posed inverse problem, which can be transformed into a sparse optimization problem. This paper uses the zero-norm as the objective function and presents a greedy algorithm called Orthogonal Matching Pursuit (OMP) to solve the corresponding minimization problem. The test results have revealed that the compressed sampling rate is approximately 14%, the standard deviation of the reconstruction error by OMP is 0.03 mGal and the signal-to-noise ratio (SNR) is 56.48 dB. In contrast, the standard deviation of the reconstruction error by the existing nearest-interpolation method (NIPM) is 0.15 mGal and the SNR is 42.29 dB. These results have shown that the OMP algorithm can reconstruct the gravity anomaly data with higher accuracy and fewer measurements.

## 1. Introduction

The Earth's gravity field is a fundamental physical field, which reflects the distribution, motion and variety of the Earth's interior matter. The gravity has connections with all the physical events on Earth and in its near space, and thus provides the basic information to support research on many subjects. Gravity surveys can support fundamental geophysical investigations, which are beneficial to determine the density of the Earth's interior matter and help explain many physical phenomena of the Earth. Meanwhile, gravity surveys are also significant in the exploitation of mineral resources and modern military science, *etc*.

Airborne gravimetry is a method of determining the Earth's gravity by using instruments on board an aircraft such as accelerometers, global navigation satellite systems (GNSS), altimeters, and attitude sensors [1,2]. Performing gravity surveys from an aircraft is superior to point-wise terrestrial gravimetry in terms of both economy and efficiency. It also offers the opportunity to measure gravity over special terrains which are difficult to access and in the areas with mixed land and ocean [3,4]. Airborne gravimetry can be applied to rapidly get extensively well-distributed information about the Earth's gravity field, so it is an important new geodetic technology and an essential part of airborne geophysical exploration and also is supplemental to terrestrial gravimetry and shipborne gravimetry.

Airborne gravimetry is essentially a discrete digital sampling method. The theoretical foundation of discrete digital sampling on continuous-time band-limited signals was developed by Nyquist and Shannon [5,6]. Their results demonstrated that signals can be recovered from a set of uniformly spaced samples taken at the so-called Nyquist rate of twice the highest frequency present in the signal of interest. However, because of the restrictions posed by national boundaries, economic cost and database size, airborne gravimetry is in practice a sub-Nyquist sampling method. Consequently, there is a question on whether the gravity data can be recovered with a new framework for signal acquisition and sensor design that enables a potentially large reduction in the sampling and computation costs for sensing signals. More recently, Candès, Romberg, Tao and Donoho showed that a signal having a sparse representation can be reconstructed from a small set of linear, non-adaptive measurements [7–11]. This result suggests that it may be possible to sense sparse signals by taking far fewer flight measurements, and hence such as a method is named Compressed Sensing (CS).

Based on airborne gravimetry technology, a flight test was carried out in China with the strap-down airborne gravimeter (SGA-WZ) developed by the Laboratory of Inertial Technology of the National University of Defense Technology [12–14]. This paper investigates a method for large scale gravity anomaly data reconstruction. Taking into account the sparsity of airborne gravimetry by DFT, this paper firstly proposes a method for the reconstruction of gravity anomaly data using the CS theory. The gravity anomaly data reconstruction is an ill-posed inverse problem, which can be transformed into a sparse optimization problem. This paper uses the zero-norm as the objective function and presents the OMP algorithm to solve the corresponding minimization problem [15]. These results have shown that the OMP algorithm can reconstruct the gravity anomaly data with higher accuracy and fewer measurements than existing methods

## 2. Basic Principle and Mathematical Model

Airborne gravimetry can be classified as airborne scalar gravimetry, airborne vector gravimetry and airborne gradient gravimetry. Our work in this paper studies airborne scalar gravimetry (we study vertical component of the vector only).

### 2.1. Airborne Gravimetry Technology

An object's gravity is the composition of forces of gravitation caused by the Earth and other celestial bodies and the inertial centrifugal force caused by the Earth's rotation. The non-uniform distribution of the density of the Earth's interior matter makes the gravity vary with the position. In gravity prospecting, the gravity variations caused by the non-uniform density distribution of the Earth's interior rocks and minerals are called gravity anomalies. In fact, the airborne gravity measurements include two parts: the gravity anomaly (denoted as the free air anomaly here) and the normal gravity (which is the reference gravity field of a conventional ellipsoid). Therefore, the gravity anomaly can be expressed as:

(1) Δg=g−γ

where Δ*g* is the gravity anomaly, *g* is the gravity measurement and *γ* is the normal gravity.

The principle of strap-down airborne scalar gravimetry is based on Newton's equation of motion in the gravitational field of the Earth, utilizing the principle of relative gravity measurement. First, we must use a terrestrial gravimeter to connect national gravity and a point on the parking apron to obtain its gravity and take it as the gravity reference point. Before the aircraft takes off, static gravimeter data must be recorded so that gravity observations in the air can be associated with the gravity reference point on the parking apron. Then, the strap-down airborne scalar gravimetry model can be written as:

(2) $$\Delta g={\dot{\nu }}_{D}-(f_{D}-f_{D}^{0}+g_{b})+\Delta a_{E}-\gamma$$

Obviously, Equation (2) is an equivalent form of Equation (1), where Δ*g* is the gravity anomaly to be determined, *ν̇_D_* is the vertical component of the vehicle acceleration obtained from GPS, *f_D_* is the vertical component of the specific force measured by the accelerometers of an inertial measuring unit, $f_{D}^{0}$ is the vertical component of the specific force obtained from the static data on the parking apron, called base reading, *g_b_* is the gravity of reference point on the parking apron and Δ*a_E_* includes all kinds of the error correction [16].

### 2.2. Mathematical Model of CS

Compressed sensing (CS) is an exciting, rapidly growing field that has attracted considerable attention in fields as diverse as electrical engineering, applied mathematics, statistics, sensor technology and computer science. CS offers a framework for simultaneous sensing and compression of finite dimensional signals. Quite surprisingly, it predicts that sparse high-dimensional signals can be recovered from highly incomplete measurements by using efficient algorithms. CS also holds promise for increasing resolution by exploiting the signal structure. Especially, reducing the sampling rate or increasing resolution in airborne gravimetry can improve survey efficiency, increase data transfer rate and improve data quality.

Let *z* be an unknown original gravity anomaly in *R^n^* (which can represent a 1-D or 2-D gravity anomaly of interest). Suppose that we have *m* linear measurements of *z* with the form:

(3) $$\begin{array}{cc} y_{i}=\langle \phi_{i},\;\text{z}\rangle +\nu_{i}, & i=1,2,\cdots ,m \end{array}$$

where 〈·,·〉 denotes the usual inner product, *y* = [*y*_1_,*y*_2_,⋯,*y_m_*]*^T^* ∈ *R^m^* is the sampling gravity anomaly, *Φ* = [ *ϕ*_1_,*ϕ*_2_,⋯,*ϕ_m_*]*^T^* ∈ *R^m^*^×^*^n^* is known and called the compressed sensing matrix and *ν* = [*ν*_1_,*ν*_2_,⋯*ν_m_*]*^T^* ∈ *R^m^* is the noise. Standard reconstruction methods require at least *n* samples. Suppose we know *a priori* that *z* is compressible or has a sparse representation in a transform domain, described by the matrix *Ψ* ∈ *R^n^*^×^*^n^*. The sparse representation of *z* can be written as:

(4) x=Ψz

where *x* is the sparse coefficient matrix and *Ψ* is the sparse transform matrix. In this case, if vectors *ϕ_i_* are well chosen, then the number of measurements *m* can be dramatically smaller than the size *n* usually considered necessary [11,17].

When *Ψ* is invertible, CS exploits the sampling gravity anomaly data reconstruction by solving a problem of the form:

(5) $$\begin{array}{ccc} \text{minimize}\;{\left\|x\right\|}_{0} & s.t. & y=\Phi \Psi^{-1}x \end{array}$$

where ‖*x*‖_0_ = |{*i*:*x_i_* ≠ 0}| is the zero-norm of *x* and denotes the number of nonzero elements within *x*. Suppose *x* has at most *k* nonzero elements, *i.e.*, ‖*x*‖_0_ ≤ *k*, we can consider that *z* is *k*-sparse in the transform domain *Ψ*.

### 2.3. OMP Algorithm

Let *A* = *ΦΨ*^−1^ ∈ *R^m^*^×^*^n^*; Equation (5) can be rewritten as:

(6) $$\begin{array}{ccc} \text{minimize}\;{\left\|x\right\|}_{0} & s.t. & y=Ax \end{array}$$

Equation (6) is the zero-norm sparse optimization problem and can be solved with greedy algorithms. Greedy algorithms rely on iterative approximation of the signal coefficients and support, either by iteratively identifying the support of the signal until a convergence criterion is met, or alternatively, by obtaining an improved estimate of the sparse signal at each iteration that attempts to account for the mismatch to the measured data. Greedy algorithms are classified as greedy pursuits and thresholding type algorithms. Greedy pursuit algorithms include Matching Pursuit [18], OMP [19], Conjugate Gradient Pursuit [20], Stagewise Orthogonal Matching Pursuit [21] and Regularized OMP [22,23], *etc*.

OMP is a greedy pursuit algorithm. Given that *z* is *k*-sparse in the transform domain *Ψ*, *i.e.*, *x* has at most *k* nonzero elements and *A* = *ΦΨ*^−1^ = [*a*_1_,*a*_2_,⋯,*a_n_*] whose columns denoted by *a_i_* ∈ *R^m^*, the vector *y* = *Ax* is a linear combination of *k* columns from the matrix *A*. In the language of sparse approximation, we say that *y* has an *k*-term representation over the dictionary *A*. Therefore, OMP can be used for recovering sparse signals.

To identify the signal *x*, we need to determine which columns of *A* participate in the vector *y*. The idea behind the OMP algorithm is to pick columns in a greedy fashion. At each iteration, we choose the column of *A* that is most strongly correlated with the remaining part of *y*. Then we subtract off its contribution to *y* and iterate on the residual. One hopes that, after *k* iterations, the OMP algorithm will have identified the correct set of columns. The major advantages of OMP are its speed and its ease of implementation. The procedure of OMP can be carried out by means of the basic steps as follows [15]:

Given the matrix *A*, the vector *y* and the sparsity level *k* of the signal *x*:

Step 1: Initialize the residual *r*_0_ = *y*, the index set *Λ*_0_ = Ø and the iteration counter *t* = 1.
Step 2: Find the index *λ_t_* that solves the easy optimization problem:
  (7) $$\lambda_{t}=\arg\max_{i=1,\cdots ,n}\left|\langle r_{t-1},a_{i}\rangle \right|$$
  If the maximum occurs for multiple indices, break the tie deterministically.
Step 3: Augment the index set and the matrix of chosen atoms: *Λ_t_* = *Λ_t_*_−1_∪{*λ_t_*} and *Ω_t_* = [*Ω_t_*_−1_ *a_λ_t__*]. We use the convention that *Ω*_0_ is an empty matrix.
Step 4: Solve a least squares problem to obtain a new signal estimate:
  (8) $$s_{t}=\arg\min_{s}{\left\|y-\Omega_{t}s\right\|}_{2}={\left(\Omega_{t}^{T}\Omega_{t}\right)}^{-1}\Omega_{t}^{T}y$$
Step 5: Calculate the new residual:
  (9) $$r_{t}=y-\Omega_{t}s_{t}$$
  It is important to note that the residual *r_t_* is always orthogonal to the columns of *Ω_t_*. Provided that the residual *r_t_*_−1_ is nonzero, the algorithm selects a new atom at iteration *t* and the matrix *Ω_t_* has full column rank. For such a case, the solution *s_t_* to the least squares problem in Step 4 is unique.
Step 6: Increment *t*, and return to Step 2 if *t* < *m*.
Step 7: The estimate *x̂* for the signal *x* has nonzero indices at the components listed in *Λ_m_*. The value of the estimate *x̂* in component *λ_t_* equals the *t*th component of *s_m_*.
Step 8: The estimate for the unknown signal *z* is: *ẑ* = *Ψ*^−1^*x̂*.

## 3. Test Results and Discussion

The strap-down airborne gravimeter and the flight test results are presented in this section. By comparing NIPM for the gravity anomaly data reconstruction, the superiority of the OMP algorithm will be shown in this section.

### 3.1. Flight Test and Data Preprocessing

**Figure 1.**
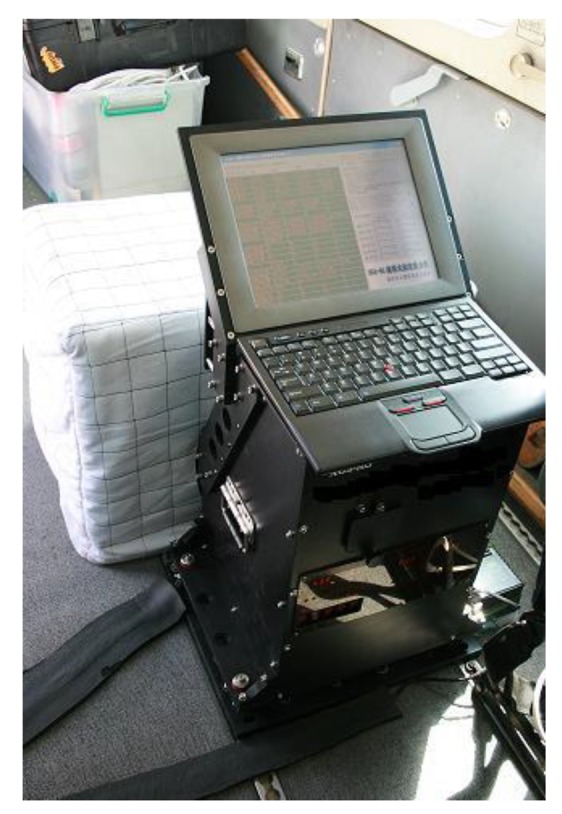
Appearance of the SGA-WZ.

The strap-down airborne scalar gravimeter called SGA-WZ mentioned in this paper is the first system with this type in China. It was developed by the Laboratory of Inertial Technology of the National University of Defense Technology [13]. This system consists of a high-performance strap-down inertial navigation system (SINS), a GNSS receiver, an anti-vibration system, a data logger and post-processing software. The major advantage of this system is its reliability and robustness in operations. A photograph of the system is shown in Figure 1.

**Figure 2.**
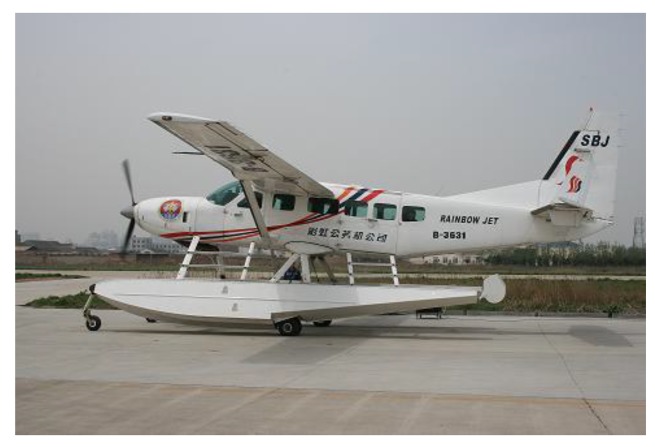
Cessna 208 fixed-wing small aircraft.

The flight tests were carried out in Shandong Province using SGA-WZ from April 2010 to May 2010. The hardware was installed in the aircraft six days before the flight tests. Two GNSS ground stations were located near the airport where the aircraft took off and landed. GNSS receivers installed on the ground and aircraft can be used to determine the vehicle position, velocity, and acceleration. The strap-down airborne scalar gravimeter onboard a Cessna 208 aircraft was used to collect the data. Figure 2 shows the Cessna 208 aircraft, which is a fixed-wing small aircraft.

**Figure 3.**
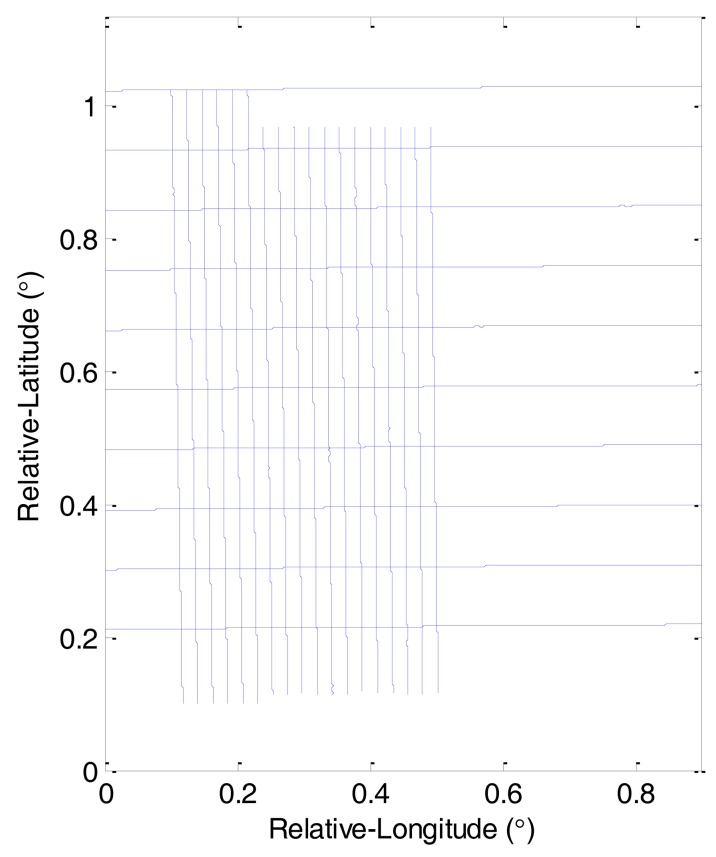
Grid flight lines.

The pilots controlled the aircraft with the autopilot, and the test was implemented in days with good weather to minimize the effects of air turbulence. The average flight altitude was approximately 400 m above sea level with a fluctuation of 20 m. The average speed during the flight was 60 m/s. The sampling rate of raw SINS readings was 100 Hz and 2 Hz for the GNSS sampling rate. After being installed in the aircraft, SGA-WZ worked all day for over a month. In the whole flight test campaign there were eight flights. The first and second flights were repeated lines that flew along the same trajectory to test the repeatability of the system, and the valid length of each repeated profile was about 100 km. The other six flights left were grid flights consisting of three flights of survey lines and three flights of control lines. The spacing between survey lines was about 2 km and the spacing between control lines was about 9 km. Figure 3 shows the grid flight lines used in the test [16].

**Figure 4.**
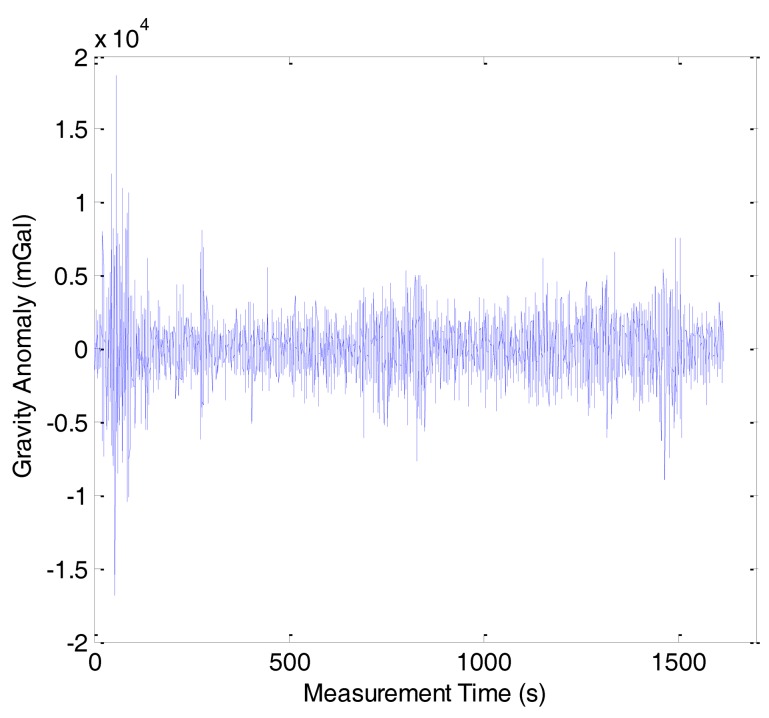
Gravity anomaly before low-pass filtering.

**Figure 5.**
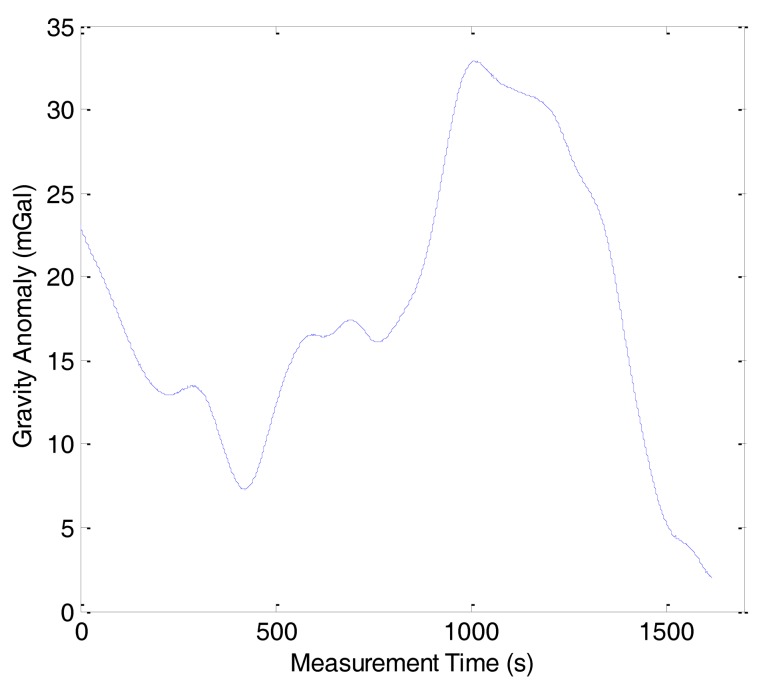
Gravity anomaly after low-pass filtering.

As previously mentioned, the gravity anomaly was determined from the difference between the specific force and the vehicle acceleration using Equation (2). Because of the phugoid modes of the aircraft and the atmospheric turbulence, there were many high frequency noises in the gravity anomaly. The noises should be eliminated by the FIR low-pass filter. When the velocity of aircraft is fixed, the longer the filter length, the more high frequency noises can be eliminated, but the spatial resolution will be lowered. The test results have revealed that the internal accord precision is 1.50 mGal with a spatial resolution of 6 km. Figure 4 shows the gravity anomaly of line 1 in the first flight before low-pass filtering. Figure 5 shows the gravity anomaly after 160 s cutoff low-pass filtering.

### 3.2. Sparsity Analysis

**Figure 6.**
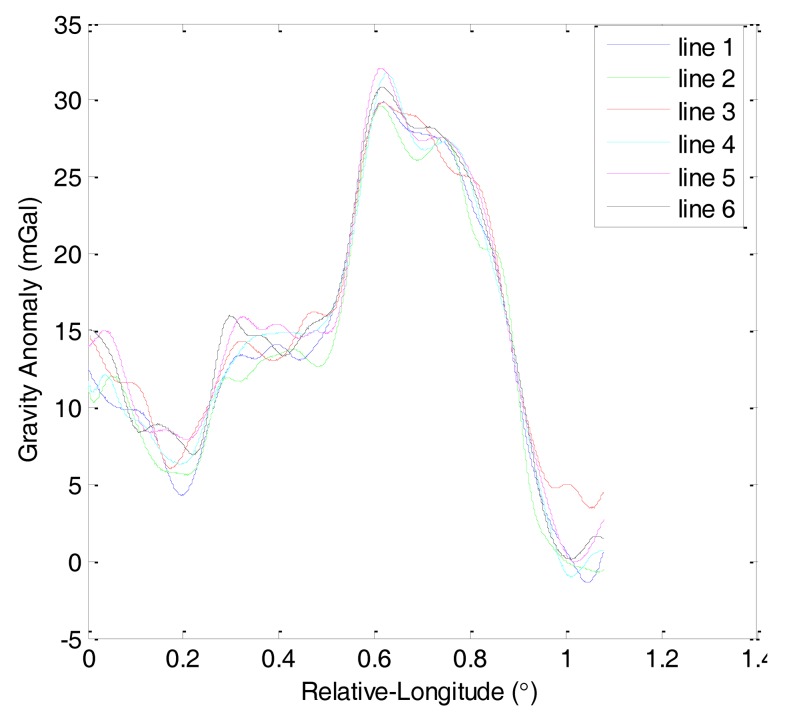
Gravity anomaly curves of F401.

**Figure 7.**
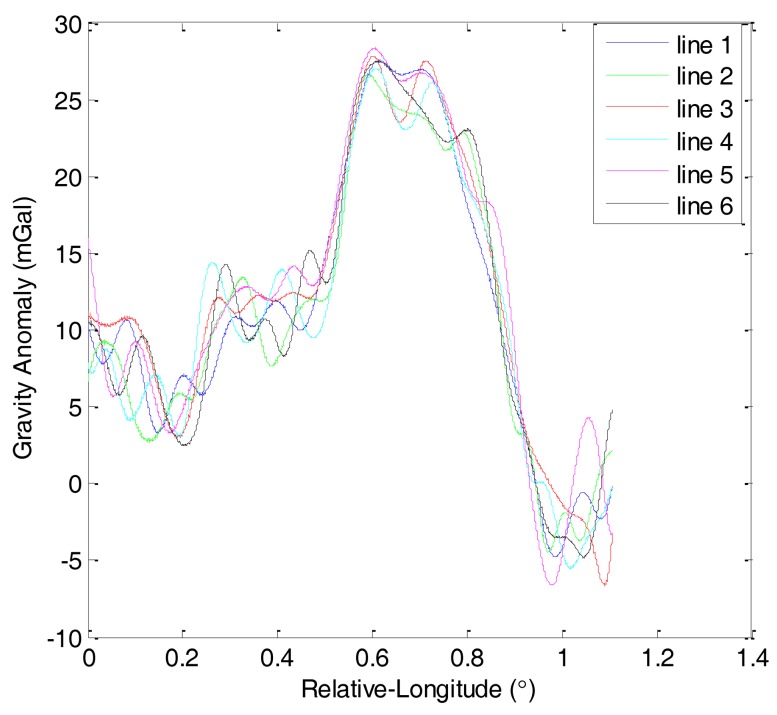
Gravity anomaly curves of F402.

The first and second flights were repeated lines surveys denoted by F401 and F402, respectively. Each of them consisted of six lines. Figure 6 shows the gravity anomaly curves of F401 and Figure 7 shows the gravity anomaly curves of F402. In the two flights, the repeated lines were surveyed along an east-west direction.

**Figure 8.**
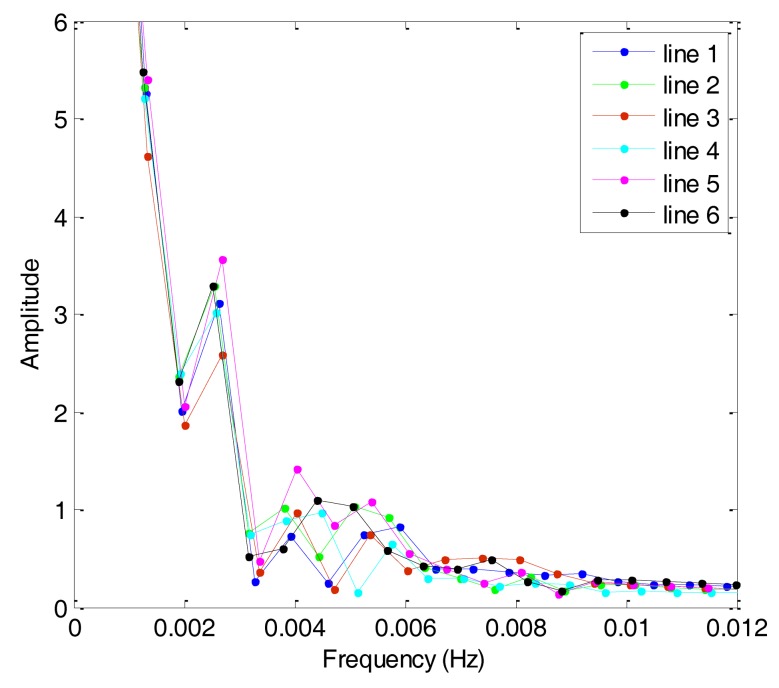
Amplitude spectrum curves of F401.

**Figure 9.**
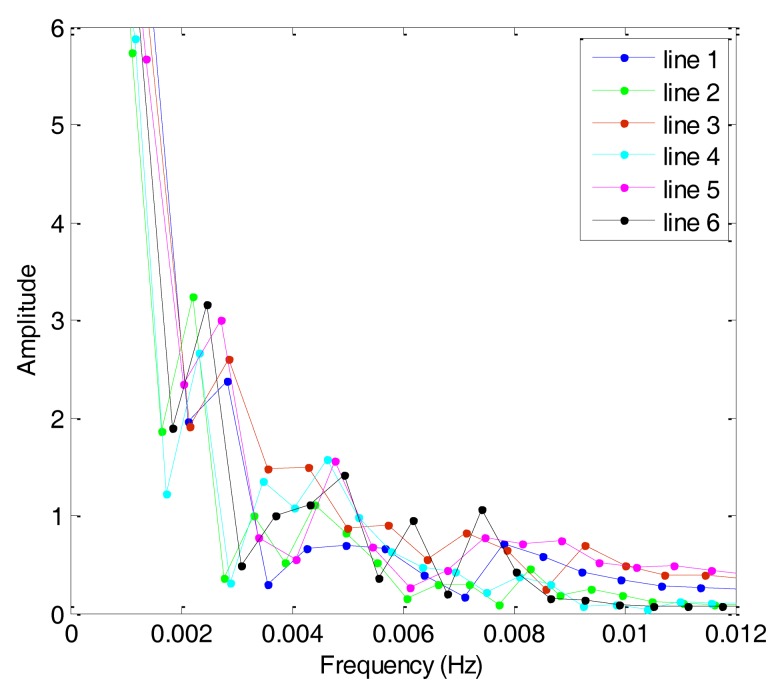
Amplitude spectrum curves of F402.

Based on the theory of CS, this study analyzes the sparsity of airborne gravimetry by DFT. Figure 8 shows the amplitude spectrum of all lines in F401 and Figure 9 shows the amplitude spectrum of all lines in F402. From the two figures, we can see that the gravity anomaly is mostly distributed in the low-frequency domain and it has few high-frequency components. The results thus reveal that the gravity anomaly is sparse in the DFT domain, which satisfies the precondition for application of CS.

### 3.3. OMP Reconstruction Results

To evaluate the OMP algorithm performance and the gravity anomaly data reconstruction precision, we now define the performance indices with standard deviation and SNR. SNR can be calculated by:

(10) $$\text{SNR}=20\log_{10}({\left\|z\right\|}_{2}/{\left\|z-ẑ\right\|}_{2})$$

**Figure 10.**
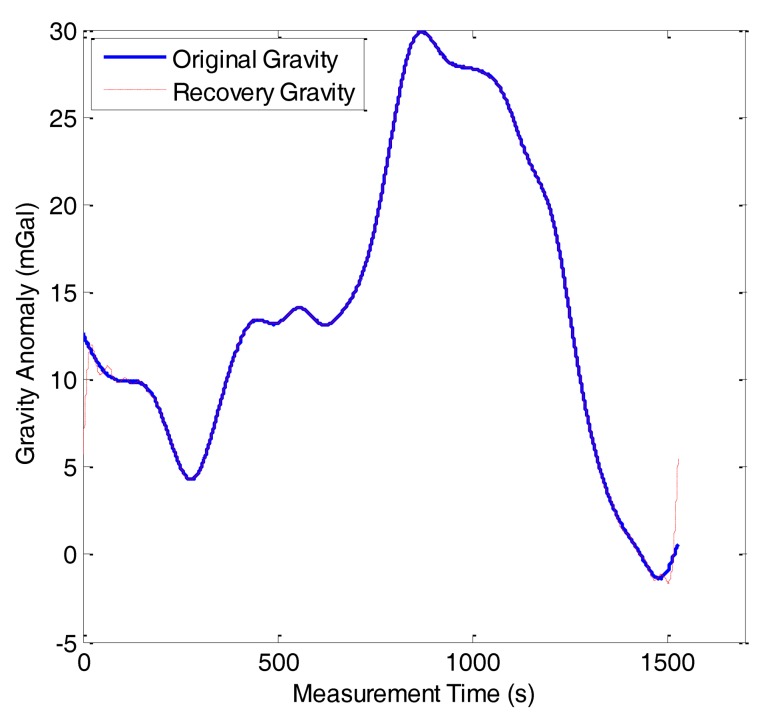
Reconstruction result of OMP in F401.

**Figure 11.**
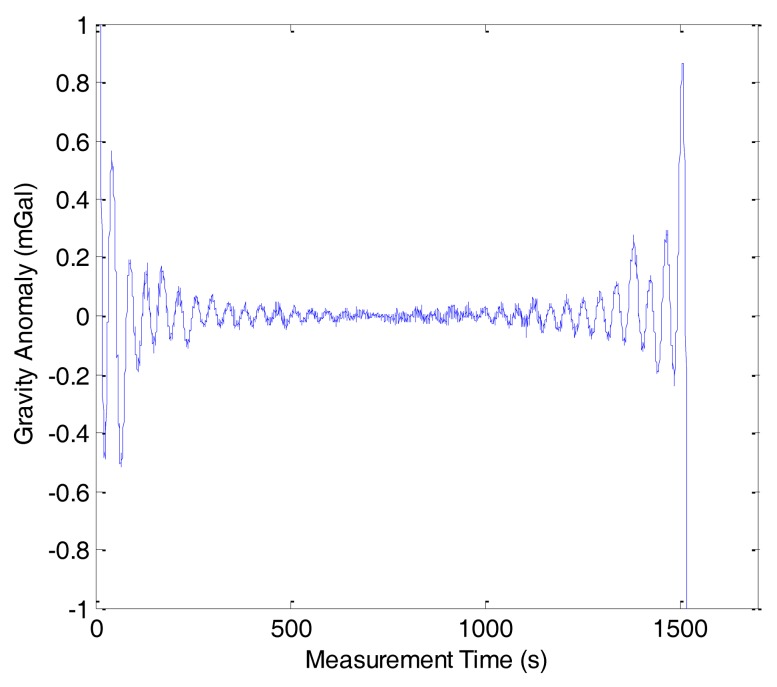
Reconstruction error of OMP in F401.

In this study, the compressed measurement number was set as *m* = 432, *i.e.*, the compressed sampling rate was approximately 14%. Figure 10 shows the reconstruction result of line 1 in F401 and the reconstruction error is shown in Figure 11. In Figure 10, the red dashed line denotes the reconstruction gravity anomaly and the blue solid one denotes the original gravity anomaly. The two figures show that the OMP results approximate the original gravity anomaly very well and the reconstruction error is very small. However, we also note that a boundary effect exists in the result and this problem should be solved in the future.

**Figure 12.**
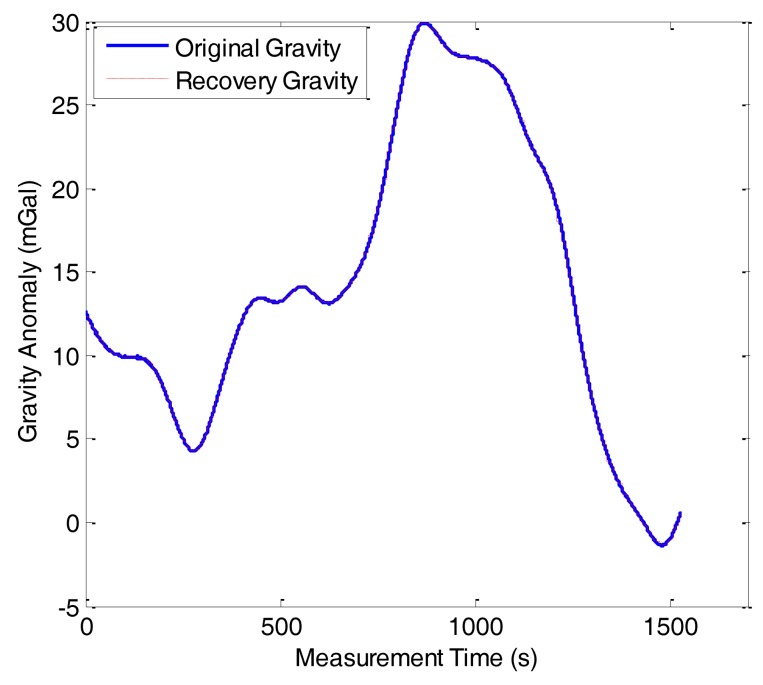
Reconstruction result of NIPM in F401.

**Figure 13.**
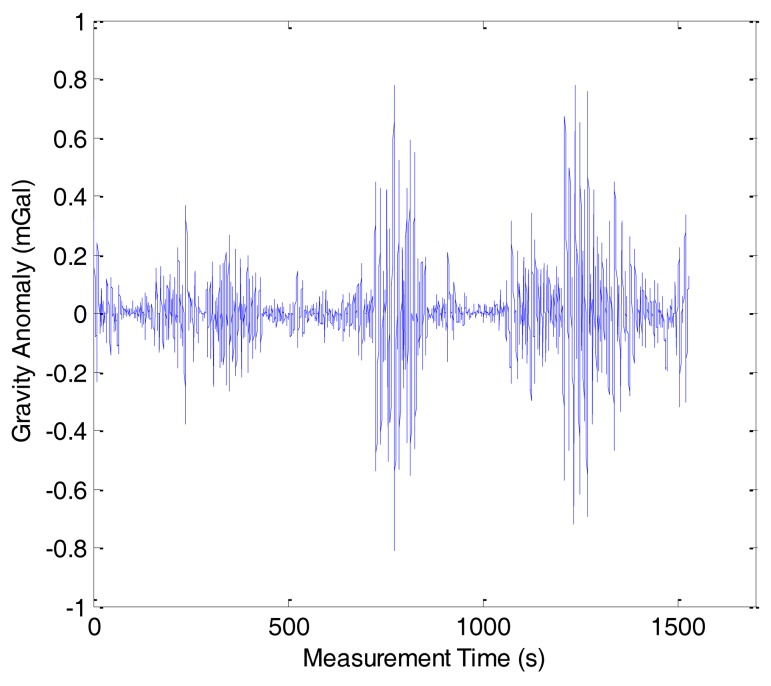
Reconstruction error of NIPM in F401.

For comparison purposes, we also made the gravity anomaly data reconstruction with NIPM, which is one of the commonly used methodologies for data reconstruction. Figure 12 shows the reconstruction result of line 1 in F401 and the reconstruction error is shown in Figure 13. The comparison of the algorithm performance between OMP and NIPM is shown in Table 1. It shows that the each performance index from OMP is superior to the corresponding one from NIPM. The standard deviation of the reconstruction error and SNR of OMP are 0.03 mGal and 56.48 dB, respectively. Correspondingly, the standard deviation of reconstruction error and SNR of NIPM are 0.15 mGal and 42.29 dB, respectively.

## 4. Conclusions

This paper has presented a new investigation on a method for high accuracy large scale gravity anomaly data reconstruction. Based on the airborne gravimetry technology, a flight test was carried out in China using a custom designed strap-down airborne gravimeter. Taking into account the sparsity of airborne gravimetry by DFT, this paper proposes a method for gravity anomaly data reconstruction using CS theory. The test results have revealed that the compressed sampling rate is approximately 14%, the standard deviation of the reconstruction error by OMP is 0.03 mGal and SNR is 56.48 dB. In contrast, the standard deviation of the reconstruction error by NIPM is 0.15 mGal and SNR is 42.29 dB. These results have shown that OMP can reconstruct the gravity anomaly data with higher accuracy and fewer measurements. In future investigations, the following considerations should be taken into account:

(1) The boundary effect exists in the result of gravity anomaly data reconstruction with OMP. Although we can ignore the boundary data, that will result in a waste of data and increase the survey costs.
(2) The discrete Fourier transform was effective for the 1-D gravity anomaly data sparse transform in this study. In consideration of the 2-D gravity anomaly data reconstruction, future work can pay attention to the Curvelet transform.

## Figures and Tables

**Table 1.** The comparison of the algorithm performance for F401.

**Number**	**Points**	**Standard Deviation (mGal)**		**SNR (dB)**	

		OMP	NIPM	OMP	NIPM
Line 1	3050	0.03	0.15	56.91	42.06
Line 2	3150	0.03	0.16	55.76	41.03
Line 3	2979	0.02	0.14	58.15	42.81
Line 4	3120	0.03	0.14	56.65	43.00
Line 5	2964	0.03	0.16	57.08	42.23
Line 6	3170	0.04	0.15	54.31	42.60
Average	3072	**0.03**	**0.15**	**56.48**	**42.29**
